# A qualitative study of challenges and enablers faced by private general practitioners providing primary care to patients with complex needs in Singapore

**DOI:** 10.1186/s12875-022-01625-x

**Published:** 2022-01-19

**Authors:** Peng Yong, Andrew Wong, Foong Yee, Sara Chan, Laysee Ong, Kheng Hock Lee

**Affiliations:** 1 Office of Community Engagement and Education, SingHealth Community Hospitals, SingHealth Tower, 10 Hospital Boulevard, Singapore, 168582 Singapore; 2 Duke-NUS Medical School, 8 College Rd, Singapore, 169857 Singapore

**Keywords:** General Practitioners, Complex care, Primary Care, Enablers, Challenges, Solutions

## Abstract

**Background:**

Singapore faces an ageing population with increasingly complex healthcare needs, a problem which could be addressed by high quality primary care. Many patients with complex needs are not managed by private general practitioners (GPs) who form the majority of the primary care workforce. Currently, there is paucity of literature describing the needs of these private GPs in providing such care.

**Aim:**

Understand the challenges, enablers and possible solutions from the perspective of private GPs in providing primary care of patients with complex needs.

**Method:**

We conducted a qualitative study using an inductive approach. Private GPs were interviewed using a semi-structured question guide with convenience sampling until thematic saturation was reached. These 12 interviewees were part of a network of clinics that provide primary care for complex patients who were recently discharged from a community hospital providing post-acute care. Data was transcribed prior to a process of familiarisation, coded and analysed using thematic analysis by three independent investigators.

**Results:**

Three themes emerged in the analysis. From a micro-organizational standpoint, private GPs and patients with complex needs must be willing to accept each other to have a therapeutic encounter (e.g., patients’ multidimensional needs, GP clinic set-up is simple yet busy). Next, from a meso-organizational view, trust and good communication channels between the referring doctors and private GPs must exist for effective collaboration in managing complex care. Lastly, macro-organizationally, external stakeholders (e.g., policy-makers) should fund care models, which are financially viable to both patients, and private GPs (e.g., via adequate subsidies and renumeration respectively) as such complex care require many resources.

**Conclusion:**

Multiple factors exist which influence the ability of private GPs in Singapore to care for patients with complex needs. Addressing these factors may reduce the over dependence on high-cost hospitals for care delivery in similar healthcare systems.

## Introduction

Singapore’s population is ageing rapidly. The proportion of elderly has risen from 9% in 2010 to 15.2% in 2020 [1] and approximately 40% of such elders have at least three chronic diseases [2]. Multiple chronic diseases, especially complex diseases, are linked to increased mortality and healthcare utilization [3]. One solution is to have high quality primary care. The National Academy of Sciences, USA has defined this as “the provision of whole-person, integrated, accessible and equitable healthcare by inter-professional teams who are accountable for the majority of an individual’s health and wellness needs across settings and through sustained relationships with patients, families and communities” [4]. Studies show that such primary care has consistently resulted in better health outcomes, reduced hospitalizations and visits to the emergency department [5].

In Singapore, primary care is provided by private general practitioner (GP) clinics and publicly funded polyclinics, similar to its tertiary counterpart in private and public restructured hospitals respectively. Private GPs usually practice singly or in groups in housing estates and workplaces. Their practices operate on a high cost, as they have to pay the prevailing market rental and other business costs [6]. Elders with multiple co-morbidities tend to pay significantly higher out-of-pocket fees here, especially for medication and ancillary services (e.g., physiotherapy), despite available yet limited government subsidies (e.g., Community Healthcare Assist Scheme (CHAS), Pioneer Generation (PG) Scheme [7]). They would often visit polyclinics to enjoy greater subsidies [8] for a wider range of services and a larger repertoire of medications, made available through bulk purchases from government linked logistic suppliers. This is supported by 2014’s figures [9] where 29% of their attendees were elderly, compared to 11% in private GP clinics. Likewise, 52% of visits to polyclinics were for chronic diseases, compared to 20% in private GP clinics.

Not surprisingly, there are long waiting times at specialist outpatient clinics in public hospitals in Singapore [10] and the hospitalization rates of the elderly remained high at 342 per 1000 resident population in 2017 [11]. This implies that their health needs are not met through primary care providers in the community, resulting in public hospitals remaining as the center of care for those with complex conditions. Complex conditions, though traditionally defined as two or more diseases which may influence the care of other conditions [12], certainly goes beyond the number of chronic diseases one has. Outside Singapore, Canadian GPs see complexities that encompass medical, mental and social domains, worsened by a poorly resourced and uncoordinated healthcare system [13].

One reason for the over-utilization of tertiary healthcare sources by the elders in Singapore is that the potential of private GPs in providing complex care is not fully expended, due to various obstacles. Lai et al. [14] stated that patients and caregivers felt that the obstacles lie mainly in the high healthcare cost outside the public system. Similarly, Foo mentioned that polyclinic doctors, caregivers and patients cited inadequate access, delivery of timely care and patient’s self-management of multiple chronic problems as obstacles in holistically managing such patients [15] - many of these may also be issues faced by private GPs. A knowledge gap exists in current literature, as private GPs in Singapore were not interviewed for their opinion of these barriers, even though they form the majority of primary care providers locally. Their perspective would shed greater insight on how to maximize their potential in managing complex care. This may be applicable to countries with similar primary care situations as there’s paucity of such studies in private GP settings.

Thus, we sought to address this gap by seeking the views of private GPs regarding the enablers, stumbling blocks and possible solutions that could anchor the care of patients with complex needs in the private GP setting.

## Methods

### Participants and Settings

We conducted a qualitative descriptive study using an inductive approach and reached out to private GPs involved in an existing care transition program in Singapore. Initialized in 2017, this program (Integrated Primary Care of At-Risk Elderly or (iPCARE) in short) helps patients with complex medical needs to transit back into the community [16]. Under this program, a network of private GPs volunteered to provide primary care for recently discharged patients with the support of multidisciplinary care and case management provided by the community hospital. (Community hospitals provide rehabilitative and convalescent care to patients who are unable to return home after an admission to an acute hospital.) The patients managed under this program were aged 60 and above and reside in the northeast region of Singapore.

These GPs are highly motivated to provide high quality primary care, knowing full well the challenges that they might face. Obtaining the opinions and insights of private GPs would help us understand the challenges and potential solutions to the problem of providing high quality primary care to patients with complex needs. They have the actual experience of providing primary care to patients with complex needs within the context of the current healthcare system and its prevailing policies. There are 69 potential private GPs in this programme (convenience sampling). The investigators randomly invited one GP at a time via either email or phone and interviewed them individually until thematic saturation was achieved. We invited 13 GPs in total, of which one declined because of conflict with meeting time.

### Data collection

We conducted these 12 in-depth interviews (IDIs) in English in the privacy of the GP’s consultation rooms, using a semi-structured question guide. The semi-structured guide was developed to enable a constructivist approach during data collection. The questions are:

1. What is the profile of complex patients who can potentially be cared for by private GPs in the community?
2. What are some factors that has enabled successful care transition and continuing care with private GPs in the community?
3. What challenges do you face in the care of such patients?
4. In your opinion, what could be done to better improve the process of care transition and continuing care of complex patients from the hospital to your clinic?

Participants were encouraged to speak freely and raise issues important to them. The interviews generally lasted between 45 to 75 min each and were audio-recorded. Fieldnotes were also recorded during the interview. Anonymity was maintained by assigning participants with a unique serial number. The interviews continued until no further themes emerged i.e., thematic saturation was reached.

### Data analysis

All interviews were transcribed verbatim and random samples were checked against the fieldnotes for accuracy. These data were analysed on an ongoing basis using an inductive approach and thematic analysis [17]. After the investigators reviewed and were familiar with the recordings and transcripts, themes and sub-themes were identified following data organization and coding. Reliability was maximised by having all three investigators independently coding all transcripts. The codes were subsequently compared between investigators and any discordance was resolved through consensus discussions. The final themes were generated through an iterative process as the team members shared and discussed their perspectives based on the study objectives. Results of this study were emailed to all participants for confirmation (member checks) and there were no comments or amendments. Demographic information of the participants was obtained to establish generalizability of our findings.

### Reflexivity

The main investigators conducting IDIs were a family physician and a trained senior staff nurse, concurrently a case-manager. They are both experienced in transitional care and work with private GPs and patients in iPCARE. The third investigator holds a PhD in psychology and is trained in social sciences. The last investigator, a family physician, was the medical director of the community hospital and has extensive experience in transitional care and community engagement. He conceptualized the initial research question while the former three investigators assisted to code and analyze the research data. The investigators engaged in reflexivity by constantly reflecting about the influence of their work experiences on note taking during interviews, audio transcription, data coding and analysis.

### Ethics consideration

The study was approved by SingHealth Centralized Institutional Review Board (CIRB 2018/2826).

## Results

### Demographics of participants

Of the 12 GPs, 11 (91.7%) were ethnic Chinese, 10 (83.3%) were male and 9 (75%) were at least 40 years old (See Table 1). All interviewees were private GPs with at least five years working experience, 11 of them (91.7%) are accredited with government subsidy schemes for chronic disease management and 11 (91.7%) are accredited by Family Physician Accreditation Board of Singapore. There was no subsequent withdrawal of consent.

**Table 1:** Characteristics of the participants

No	Age (years)	Gender	Ethnicity	Length of general practice (years)	Nature of practice	On family medicine register	Practices in family medicine clinic	Belongs to a primary care network	On government subsidy schemes	Proportion of patients receiving complex care
1	41	Male	Chinese	6	Group	Yes	No	Yes	Yes	5%
2	56	Female	Chinese	33	Solo	Yes	No	Yes	Yes	< 5%
3	40	Male	Chinese	5	Solo	Yes	No	Yes	Yes	1.5%
4	38	Male	Chinese	8	Group	Yes	No	Yes	Yes	20%
5	44	Male	Chinese	15	Group	Yes	No	Yes	Yes	5%
6	35	Male	Chinese	6	Group	Yes	No	Yes	Yes	20%
7	53	Male	Chinese	29	Group	Yes	Yes	No	Yes	95%
8	40	Male	Chinese	5	Group	No	No	Yes	Yes	20%
9	38	Female	Chinese	7	Group	Yes	No	Yes	Yes	20%
10	40	Male	Chinese	15	Solo	Yes	No	Yes	Yes	5%
11	65	Male	Indian	30	Solo	Yes	No	No	No	40%
12	54	Male	Chinese	26	Solo	Yes	No	No	Yes	16%

### Qualitative results

Three themes emerged during the analysis and these are enumerated with their subthemes in Table 2.

**Table 2:** Themes and Subthemes

Themes	Subthemes
1. Private GPs and patients with complex needs must be willing to accept each other to have a therapeutic encounter. (micro-organisational)	a) Private GPs and such patients consider the presence of certain skill sets (e.g. geriatric training) and approach (e.g. generalist) of private GPs before coming together for consultation
	b) Private GPs and such patients factor in the clinic set-up (e.g. accessibility, presence of resources, less workload of seeing acute conditions) before engaging in consultations
	c) Private GPs and such patients consider the multi-dimensional needs of the latter (i.e. biopsychosocial domains) before engaging in consultations
2. Trust and good communication channels between the referring doctors and private GPs must exist for effective collaboration in managing complex care. (meso-organisational)	a) Patients with complex needs have multi-dimensional issues which require careful handover of details from referrer to private GPs and resources from both parties (e.g. time on the part of GPs, casemanagers from referers)
	b) Private GPs perceive themselves to be negatively evaluated (e.g. in terms of clinical skills) by the public (including the referers of patients with complex needs), resulting in less of such referrals to them
3. External stakeholders (e.g., policy-makers) should fund care models, which are financially viable to both patients with complex needs and private GPs. (macro-organisational)	a) The management of complex care needs to be affordable to such patients in private clinics as they generally require more resources
	b) The management of complex care has to be financially sustainable for private GPs as such consultations may consume more time than uncomplicated care and require highly personalised and contextualised management

#### Mutual acceptance between private GPs and patients with complex needs

Micro-organizationally, private GPs and patients with complex biopsychosocial needs must be willing to accept each other to have a therapeutic encounter, especially when some patients may not see the value of seeing a generalist.

“… Dementia patients… have significant social issues… We notice things like caregiver burden, caregiver stress and burn out… Some patients… only have one caregiver. They have financial problems. To be honest we don’t quite have that time to really dwell into it.” (Interviewee 4’s view on characteristics of complex cases which private GPs find difficult to accept)

“…Rehabilitation is not something I have main experience in, so I do turn to other people for help… Certain advice may be a bit more difficult for me, especially end of life care, advanced care planning, sometimes all the tube-feeding…I can do that but it has been some time…” (Interviewee 10’s view on characteristics of complex care which private GPs find difficult to accept)

“… So, when they have somebody (GP) tell them, oh you have very high cholesterol… you need to go on certain medications. And when they are anxious…they go the heart specialist, they’ll have extensive investigations done but the conclusion is still the same…And they have spent money needlessly. If they have the confidence in a generalist, that wouldn’t have happened….” (Interviewee 11’s view on barriers to complex care)

An interviewee stated that private GPs may likely take up more complex cases if their workload for acute consultations is reduced.

“…. the simple acutes or sometimes those who are here for MCs, if possible, to reduce that load, then we can focus more on the complex chronics who need more time…. The best thing to have is actually to allow for people to call in sick…. the next bulk will be those who are not very sick who can take medicine just from the pharmacy…” (Interviewee 3’s view on enablers/solutions to complex care)

#### Trust and good communication channels between referrers and private GPs

Secondly, trust and good communication channels between the referring doctors and private GPs are crucial for effective collaboration in managing complex care (meso-organisation). Detailed memos and real-time access to help greatly facilitates this communication.

“… we happen to be the nearest clinic, can be a bit tricky because we end up looking at the patient and not understanding why the patient is here. Patient also don’t know why they are here… If I have to look at the memo and don’t know why they are sending to me, patient also don’t know what they are doing here then I think that one is probably the worst thing that can happen.” (Interviewee 8’s view on barriers to complex care)

“… your memo to us about the case if it’s very comprehensive, explaining everything… This helps us to have more confidence to look after the patient and then…, if we have the ability to…. Contact the right-siting nurse at our convenience to ask about our patient, that also helps… so this right-siting nurse must also know the cases fairly well…” (Interviewee 2’s view on enablers/solutions to complex care)

Note: Right-siting refers to transferring the care of patients to the appropriate healthcare provider.

“… Actually, I think the FMCs (Family Medicine Centres) were quite a good initiative…there were close tie-ups between the private GPs and hospitals in proper handing over of care…” (Interviewee 4’s view on enablers/solutions to complex care)

Note: FMCs are local multi-doctor primary care clinics formed by partnering private GPs with the regional public healthcare systems so that private GPs can eventually take over the care of subsidised patients with complex needs.

Nonetheless, some private GPs feel the negative perception of the public (including the referrers of patients with complex needs) on the skill sets of private GPs, resulting in the latter’s reduced workload in complex care.

“ So what’s the work that GPs (referring to private GPs) are doing? Cough and fever … obviously right… nobody needs to see us already, so that’s the thing…” (Interviewee 6’s view on the barriers to complex care)

#### Improve funding of complex care models at private GP clinics

Lastly, external stakeholders (e.g., policy-makers) should fund care models, which are financially viable to both patients and private GPs (e.g., via adequate subsidies, renumeration for complex consultations and allowing bulk purchasing of services respectively) as such care often requires much time and resources. (macro-organisation).

“….. even if I refer them out to the community health, the community health centres, or I refer them to all the ancillary health services… they require them to go on a separate visit… that is again time wasted and cost as well…. a lot of the ancillary services are not subsidized…” (Interviewee 2’s view on barriers to complex care)

“…For example, ABCs (anonymised drug) in polyclinics are… sold to them at $1, but our cost price is not even $1…. it’s not even subsidised but they are able to get better pricing because of… bulk purchasing. Solo GPs when we get small quantity, we are not allowed to…. So that’s one way that ABC hospital tried to circumvent that….” (Interviewee 5’s view on enablers/solutions to complex care)

“… ABC hospital does it or maybe DEF right-siting program…just pay you the consultation and then they courier the medication over to the patients… The government should heavily subsidise because… if they are paying subsidised rate about $30 for patient, but the consultation takes 20–30 min, we are not profiting from the medication… the government should at least give the GP a much more competitive rate like maybe $JKL for 20–30 min consultation. I think that’s not a lot if we are spending this amount of time and we are highly trained…” (Interviewee 5’s view on solutions to complex care)

## Discussion

Our study demonstrates that private GPs in Singapore who are willing to take care of patients with complex care needs face multiple challenges in the healthcare system. These challenges need to be addressed if policymakers wish to tap into the potential of private GPs to improve the care of patients with complex needs. This could greatly contribute towards building a sustainable healthcare system to meet the rising healthcare demands brought on by an ageing population.

Although there is a paucity of studies focusing on these private GP’s perceptions of patients with complex care needs, the few existing studies corroborate with our findings. For example, one study that focused on the provision of primary care to breast cancer survivors revealed similar enabler themes such as timely communication with specialists, confidence in GPs and funding support [18]. Moreover, payment and time-constraint issues were highlighted as barriers faced by private GPs providing house-calls [19]. Locally, a study looking into the public primary care system using the chronic care model identified high patient load and inadequate communications with specialists as the main challenges faced [15]. Finally, a systematic review of qualitative studies on private GPs caring for patients with multimorbidity found that the primary-secondary divide, high workload from acute medicine, and information discontinuity across settings hampers private GPs’ care of complex cases [20].

Some results in our study may differ from studies involving GPs of other nationalities. Remuneration of private GPs, patients’ willingness to pay and subsidies of healthcare services in GP are less of an issue in countries like United Kingdom and Australia where there is universal healthcare coverage for primary care. Moreover, in these countries with great emphasis on general practice, patients are assigned to fixed primary providers who play a strong gatekeeping role to tertiary care. In contrast, locally, there’s a freedom of choice for any healthcare provider and private GPs may not be viewed by some patients to be equal partners as the specialists.

There are numerous high quality primary care models- an example is the Patient Centered Medical Home (PCMH) [21], focusing on the central figure of the primary care physician who leads his multidisciplinary team to care for his patients. PCMH exists as more than a physical space- it is a medical home whose patients could return for help and an organisation fulfilling the seven principles of primary care. The principles being 1) having a personal physician 2) he/she directs overall care 3) he/she coordinates care across the entire healthcare system 4) he/she is responsible for all the care needs of the patients 5) such care is easily accessible 6) his/her team is committed to service and clinical quality improvement and safety 7) there is adequate payment for clinical services which commesurate with the efforts of the providers [22]. These principles overlap with the competencies of family physicians outlined in the constitution of the College of Family Physicians, Singapore [23]. This model indeed suits the complex care needs of most ageing populations and addresses the themes in our paper (e.g. mutual acceptance of both patient and physician in the therapeutic alliance, effective communication of stakeholders for effective care collaboration and adequate funding for healthcare). The latter is also emphasized in Rich’s white paper to the U.S department of Health and Human Sciences [24] calling for adequate payment and management of fragmented finances schemes of healthcare. This applies to similar healthcare systems such as Malaysia, where financial support for multidisciplinary resources (e.g., pharmacists) is also an important issue for private GPs [25].

Thus, the findings of our study may be helpful towards informing efforts to improve primary care in our local context. Firstly, we hope that this study informs private GPs of the various enablers to provide primary care to patients with complex care needs. Being aware of these solutions could be a source of encouragement to private GPs that they are not alone in the journey toward providing adequate primary care service to complex patients. Secondly, we can better understand the consequences of the fragmentation of primary care between the public and private sectors and work towards a unified primary care that can improve the care of patients with complex care needs. Thirdly, we can start working on ways to enlist the capacity of private primary care providers, exchanging best practices, utilizing technological advancements and reaffirming our mutual commitment to patient care.

The strength of our study lies in the ability to recruit interviewees who are experienced and actively engaged in providing care to patients with complex care needs. Secondly, the investigators are experienced and knowledgeable in the subject matter, and were able to engage and seek clarification of views of the participants during data collection. At the same time, we were able to attend to potential bias during interpretation of the themes by having investigators with diverse professional backgrounds.

There are limitations to this study which affect the generalisability of the findings to other GPs. The private GPs from iPCARE practiced mainly in the north-eastern part of Singapore, some of which are relatively new housing estates with a slightly younger population compared to other regions. Thus, there is a possibility that the perspective of the participants may not be similar to private GPs with a different practice profile. Moreover, female GPs may be unrepresented in our study and they may possess slightly different practice experiences (e.g., caring for more female elders and caregivers whose needs may differ from their male counterparts). Lastly, the private GPs in this study are motivated to provide care for patients with complex needs. Their perspective may also be different from other private GPs who did not participate in this study.

## Conclusion

Multiple factors exist which influence the ability of private GPs to care for patients with complex needs. Addressing these factors may reduce the over-dependence on high-cost hospitals for care delivery and enable private GPs in Singapore to maximise care for these patients.

## Data Availability

The data used and analysed during this study are available from the corresponding author on reasonable request.
